# Mast Syndrome Outside the Amish Community: *SPG21* in Europe

**DOI:** 10.3389/fneur.2021.799953

**Published:** 2022-01-17

**Authors:** Matthias Amprosi, Elisabetta Indelicato, Wolfgang Nachbauer, Anna Hussl, Claudia Stendel, Andreas Eigentler, Constanze Gallenmüller, Sylvia Boesch, Thomas Klopstock

**Affiliations:** ^1^Department of Neurology, Center for Rare Neurological Movement Disorders, Medical University Innsbruck, Innsbruck, Austria; ^2^Department of Neurology, Friedrich-Baur-Institute, Ludwig-Maximilians-University Munich, Munich, Germany; ^3^German Center for Neurodegenerative Diseases (DZNE), Munich, Germany; ^4^Munich Cluster for Systems Neurology (SyNergy), Munich, Germany

**Keywords:** MAST syndrome, SPG21, hereditary spastic paraplegia, rare diseases, HSP

## Abstract

**Background:**

Mast syndrome is a rare disorder belonging to the group of hereditary spastic paraplegias (HSPs). It is caused by bi-allelic mutations in the *ACP33* gene, and is originally described in Old Order Amish. Outside this population, only one Japanese and one Italian family have been reported. Herein, we describe five subjects from the first three *SPG21* families of German and Austrian descent.

**Methods:**

Five subjects with complicated HSP were referred to our centers. The workup consisted of neurological examination, neurophysiological and neuropsychological assessments, MRI, and genetic testing.

**Results:**

Onset varied from child- to adulthood. All patients exhibited predominant spastic para- or tetraparesis with positive pyramidal signs, pronounced cognitive impairment, ataxia, and extrapyramidal signs. Neurophysiological workup showed abnormal motor and sensory evoked potentials in all the patients. Sensorimotor axonal neuropathy was present in one patient. Imaging exhibited thin corpus callosum and global brain atrophy. Genetic testing revealed one heterozygous compound and two homozygous mutations in the *ACP33* gene.

**Conclusion:**

Herein, we report the first three Austrian and two German patients with *SPG21*, presenting a detailed description of their clinical phenotype and disease course. Our report adds to the knowledge of this extremely rare disorder, and highlights that *SPG21* must also be considered in the differential diagnosis of complicated HSP outside the Amish community.

## Introduction

Hereditary spastic paraplegias (HSPs) are a heterogeneous group of genetic disorders sharing a core phenotype of progressive spastic paraparesis. They are divided into pure and complicated forms, the latter being characterized by additional symptoms such as ataxia, extrapyramidal symptoms, cognitive impairment, seizures, and peripheral nervous system involvement (1, 2). Estimated prevalence rates for all HSPs range from 2 to 10/100,000. Few subtypes make up the majority of all cases, while several genotypes are rare and only occur in isolated populations (3, 4). To the latter group belongs the Mast syndrome (*SPG21*), a complicated HSP accompanied by dementia, extrapyramidal and cerebellar signs. Mast syndrome is caused by bi-allelic mutations in the *ACP33* (*SPG21*; *HSP-ACP33*) gene, coding for a protein called maspardin (5). Maspardin has been shown to localize to membranes of the *trans-*Golgi/endosomal network and the cytoplasm, and interact with the aldehyde dehydrogenase ALDH16A1 (4, 5). Disease-causing mutations result in the absence of functional maspardin. Mast syndrome was first described in the Old Order Amish population (6). Outside the Amish population, one Japanese case and one Italian family have been reported (3, 7, 8). We report the first three families with Mast syndrome in German- speaking countries.

## Materials and Methods

Three subjects from two unrelated families (families I and II) were referred to the “Center for rare neurological diseases” at the Medical University Innsbruck in Austria. Another two subjects from one family (family III) were seen at the Friedrich-Baur-Institute in Munich, Germany. The German family was mentioned in a previous report (3) but was not described in detail. All the patients were examined by movement disorder specialists. Additional investigations included brain magnetic resonance imaging (MRI), neurophysiological, neuropsychological, and laboratory examinations. Subject I-1 first presented at our department 15 years ago and is still being followed up on a regular basis. Subjects from family II were seen from 1999 (II-1) to 2012 (II-2), and have since been lost of follow-up. Subjects from family III have already passed away. Due to the particular pedigrees of our families we do not expect any other patients from these families to present at our clinics.

Family I was tested for mutations in the *PANK2, HTT*, and *ATN1* genes. Families I and II were then tested *via* HSP panels by a certified external laboratory (Centogene, Rostock, Germany). Family III was examined by whole exome sequencing (HiSeq2000 system, Illumina; average coverage 122x).

Investigations and assessments were performed on a routine clinical basis and in accordance with the Declaration of Helsinki on ethical principles for medical research. Hereafter, we will use Roman numerals to identify families and Arabic numerals for individuals.

## Results

### Case Descriptions

#### Family I

**Figure 1:**
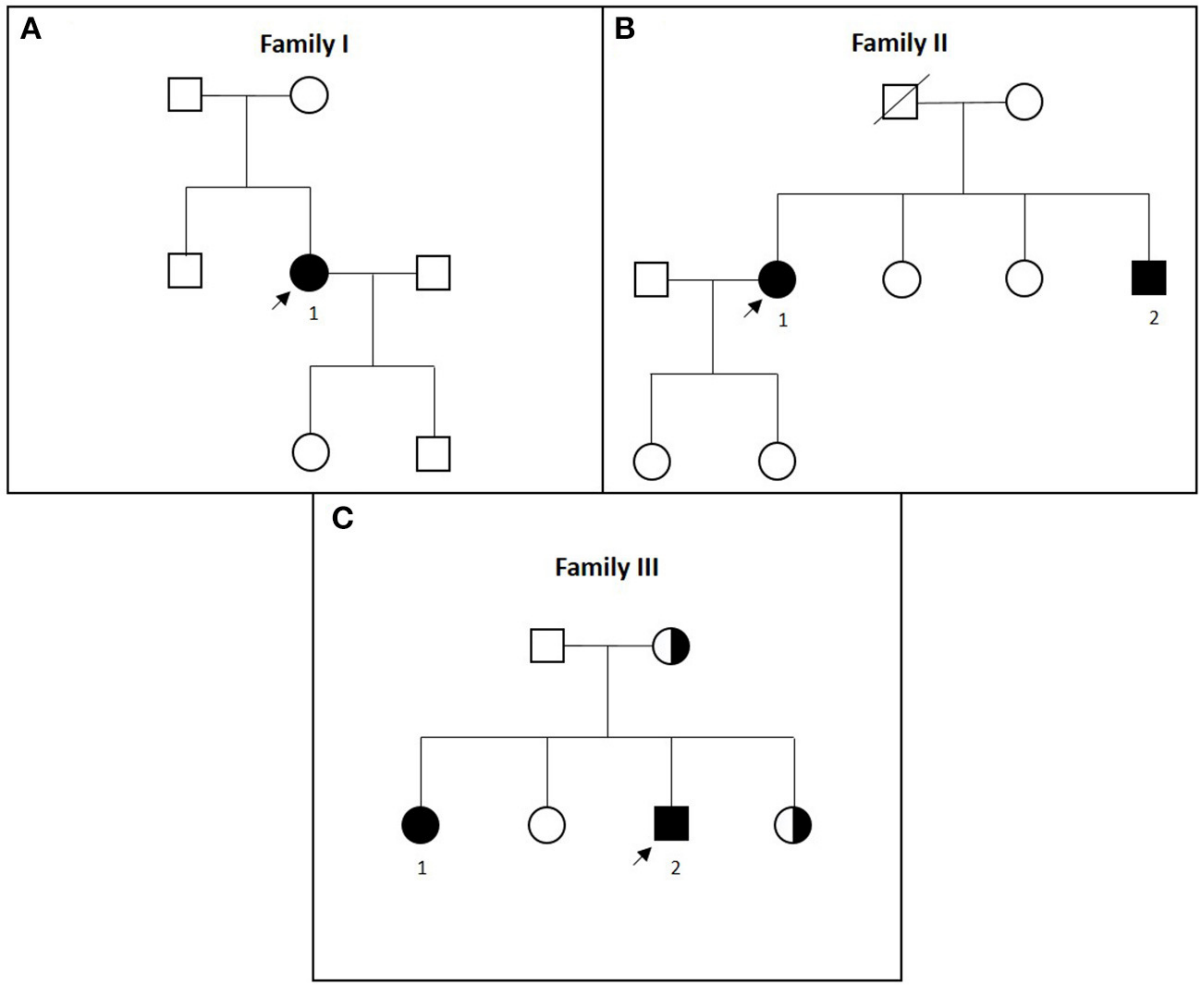
**(A–C)** Pedigrees of families I to III in chronological order. Affected patients are marked with black symbols and numbers, tested carriers are marked with half-black symbols, and index patients are marked with arrows.

Family I, which originated from Tyrol, contained one affected individual (I-1) (Figure 1A). This patient reported of always having an unsteady gait, which started to worsen at the age of 35 with increasing stiffness and muscle cramps. In the next years, she developed cognitive impairment, lost ambulation, and became incontinent. On last examination at the age of 52, the patient was wheelchair-bound, showed severe spastic tetraparesis, oromandibular dyskinesias, dysarthria, saccadic ocular pursuit, and limb ataxia, as well as pronounced dementia. Neuropsychological testing revealed a globally reduced cognitive performance. Mini-Mental State Examination showed a decrease from 20 to 6/30 points within 6 years. Brain MRI revealed thin corpus callosum (TCC) and marked global atrophy. Motor evoked potentials (MEPs) showed decreased conduction velocity in all extremities, while sensory evoked potentials (SEPs) were abnormal in the lower limbs. Moreover, peripheral dysfunction in terms of axonal sensorimotor neuropathy was detected (Table 1).

**Table 1:** Demographics and clinical characteristics of the patients with *SPG21*.

	**Subject I-1**	**Subject II-1**	**Subject II-2**	**Subject III-1**	**Subject III-2**
Mutation	Homozygous c.487delA mutations	Compound heterozygous c.118C>T, c.153delT mutations	Compound heterozygous c.118C>T, c.153delT mutations	Homozygous c.118C>T mutations	Homozygous c.118C>T mutations
Age at onset	35	30	10	20	40
Age at death	n.a.	n.a.	n.a.	54	52
Initial symptoms	Gait disturbance	Gait disturbance, dysarthria	Gait disturbance, cognitive impairment, dysarthria, ataxia	Psychosis	Gait disturbance, cognitive impairment
**Upper extremities**					
Spasticity	+	+	+	+	–
Weakness	+	–	–	+	–
Hyperreflexia	+	+	+	+	–
**Lower extremities**					
Spasticity	+	+	+	+	+
Weakness	+	+	–	+	+
Hyperreflexia	+	+	+	+	+
Pyramidal signs	+	+	–	+	+
**General**					
Extrapyramidal symptoms	Oromandibular dyskinesias	Choreatic movements of the UE, Dystonic posturing of fingers, and LE	Brady- and hypokinesia	n.k.	Choreathetosis
Dysarthria	+	+	+	+	+
Ataxia	+	+	+	+	+
Cognitive impairment	+	+	+	+	+
Psychiatric symptoms	+	+	–	+	–
Wheel chair-dependency	50 a	49 a	50 a	41 a	n.k.
Urinary/fecal incontinence	+/+	+/–	–/–	+/+	n.k.
**Imaging**					
Thin corpus callosum	+	+	+	n.k.	+
Cerebral atrophy	+	+	+	n.k.	+
Cerebell aratrophy	+	+	+	n.k.	+
White matter lesions	–	+	–	n.k.	–

*n.a., not applicable; n.k., not known; UE, upper extremities; LE, lower extremities*.

Genetic analysis revealed a novel homozygous mutation in exon 6 of the *ACP33* gene, leading to a premature stop codon (c.487delA; p.I163X). The allele frequency for this mutation in the non-Finnish European population was 0.0000176 in the Genome Aggregation Database (gnomAD v.2.1.1); thus, this does not represent frequent polymorphism in this population (9).

#### Family II

Family II originated from Upper Austria. Two siblings were affected (Figure 1B).

##### Subject II-1

**Figure 2:**
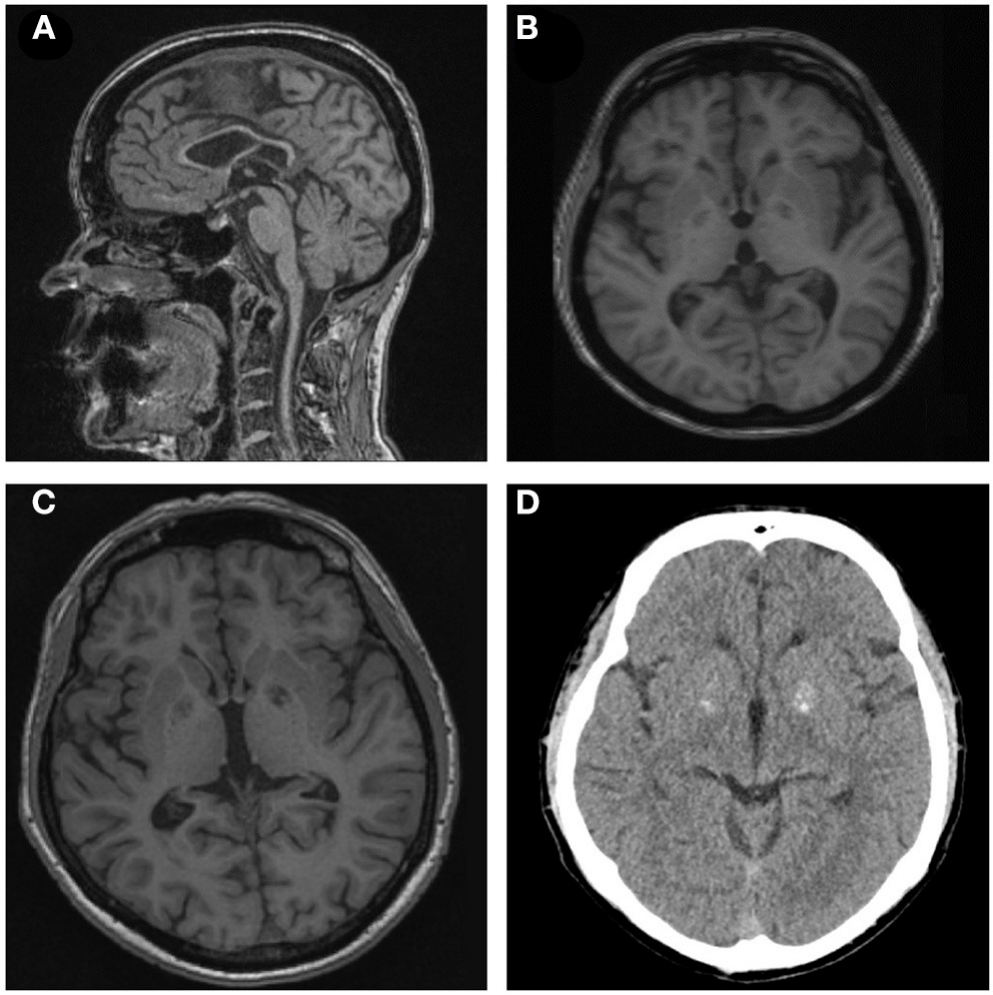
**(A,B)** Sagittal T1-weighted brain magnetic resonance imaging (MRI) of patient II-1 demonstrating thin corpus callosum. Axial T1-weighted brain MRI of subject I-1 depicting cortical atrophy. **(C,D)** T1-weighted MRI and CT scan of subject II-2 showing basal ganglia calcification.

Subject II-1 first experienced dysarthria, gait disturbances, and falls at the age of 30. Subsequently, she developed weakness and stiffness of the lower extremities, decreased dexterity, akathisia, and signs of cognitive impairment. At last follow-up at the age of 49, she was demented and non-ambulatory, and she exhibited dysarthria, saccadic ocular pursuit, hypometric saccades, limb ataxia, lower limb spasticity, choreatic movements of the upper extremities with dystonic posturing of the fingers as well as dystonic posturing of the lower extremities. Brain MRI revealed TCC, global atrophy, pronounced leukoencephalopathy, and pallidal calcifications (Figure 2). MEPs and SEPs showed decreased conduction velocity in the lower extremities. Electroencephalogram (EEG) and nerve conduction studies (NCSs) were unremarkable. Neuropsychological testing at the age of 40 revealed apraxia, impairments in visual memory, and reduced processing speed (Table 1).

##### Subject II-2

The 18 years younger brother of subject II-1 developed an unsteady gait, slurred speech, impaired dexterity, and cognitive impairment at the age of 10. The course was stable for several years but worsened at the age of 24. On last examination at the age of 36, he presented with dysarthria, saccadic ocular pursuit, hypermetric saccades, lower limb spasticity, limb ataxia, spastic-ataxic gait, brady-, and hypokinesia, and cognitive impairment. Brain MRI revealed global cerebral atrophy, TCC, and calcifications in the pallidum (Figure 2). MEPs and SEPs showed reduced central conduction velocities. NCS and EEG were normal. Neuropsychological testing was performed at the age of 30 and 36 and revealed progressive impairment in most cognitive domains (Table 1).

##### Genetic Testing

Subject II-1 was tested negative for mutations in the *PANK2* gene within the frame of a previously published study (10). Analysis for Huntington's disease and Dentatorubral-pallidoluysian atrophy was unremarkable. HSP panel analysis showed compound heterozygous mutations in exon 3 of the *ACP33* gene. One mutation (c.118C>T; p.R40X), leading to a premature stop codon, was also found in family III. The second mutation (c.153delT; p.Val52fs) is novel and causes a frameshift. Variant 1 has an allele frequency of 0.00002637, while variant 2 is not listed in the gnomAD v.2.1.1 database. Thus, neither of the variants is a frequent polymorphism (9).

#### Family III

Family III originated from Bavaria, Germany (Figure 1C).

##### Subject III-1

At the age of 20, the subject developed paranoid psychosis requiring inpatient treatment. Five years later, slowly progressive gait difficulties and progressive cognitive decline were noted. At the age of 29, mild dementia was diagnosed, accompanied by dysarthria, spastic tetraparesis, and gait ataxia. At the age of 35, speech was limited to yes/no answers and echolalia. The patient became wheelchair-bound at the age of 41 when she also presented with urinary and fecal incontinence. At last follow-up at the age of 51, she was bedridden and mutistic, and displayed multiple severe contractures (Table 1). She died at the age of 54.

##### Subject III-2

Subject III-2 complained of progressive gait difficulties, and concentration and word-finding difficulties since the age of 40. At the age of 43, he showed severe dysarthria, saccadic pursuit, square wave jerks, spastic paraparesis, limb and gait ataxia, and choreoathetosis. Fundoscopy revealed bilateral pigmentary retinopathy. Neuropsychological testing confirmed severe cognitive deficits with a total score of 10 out of 30 in the Montreal Cognitive Assessment (MoCA). Brain MRI showed generalized cerebral atrophy with frontoparietal predominance and TCC. Spinal MRI showed mild atrophy. Last follow-up at 48 years of age revealed significant worsening of the cognitive, motor, and cerebellar deficits. In addition to the clinical findings mentioned above, severe apraxia, vertical gaze palsy, and a visual acuity of 0.5 bilaterally were noted (Table 1). The patient died of unknown reason at the age of 52.

##### Genetic Testing

Both patients were homozygous for a novel truncating mutation in exon 3 of the *ACP33* gene: c.118C>T; p.Arg40^*^. One sister and their mother were unaffected mutations carriers. As mentioned above, this variant is not a frequent polymorphism in the gnomAD v.2.1.1 database (9).

## Discussion

We describe the first three Austrian and German families with complex HSP due to previously unreported mutations in the *ACP33* gene.

**Figure 3:**
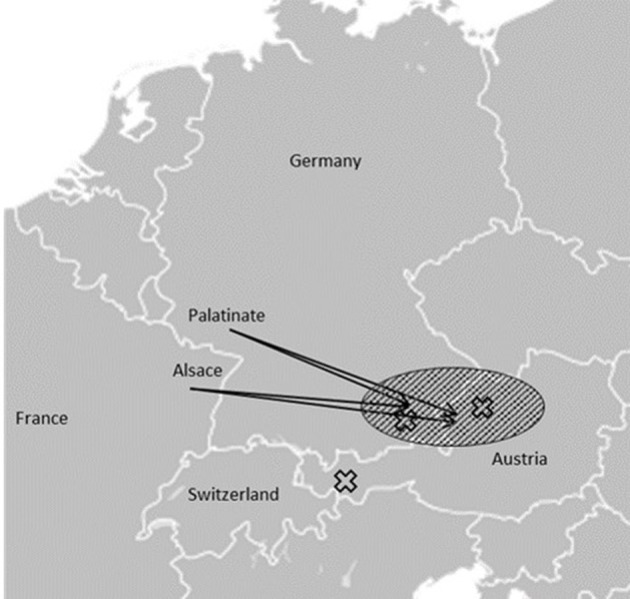
Schematic representation: Amish movements from the Palatinate and Alsace regions to Bavaria and Upper Austria in the late eighteenth and early nineteenth centuries are marked with arrows and a dashed field. The origins of the reported families are marked with X, families II and III originated from within the area of Amish settlements (Figure is based on “Blank map of Europe.svg” by Maix which is licensed under CC BY-SA 3.0).

The first cases of Mast syndrome reported by Cross and McKusick were found within the Older Order Amish in Ohio, United States (6). Causative frameshift mutation in the *ACP33* gene led to a premature stop codon, likely causing loss of function (4). The Amish Order, after being founded in Switzerland at the end of the seventeenth century, migrated throughout Europe with settlements in the Alsace and Palatinate regions. From there, they moved to different parts of Europe, including Bavaria and Austria, as well as to the United States where they founded the Old Order Amish settlements (11). None of the reported families here harbor the Amish founder mutation. Families II and III, however, originate from areas in geographical proximity of the German/Austrian border (Figure 3) and share the same c.118C>T mutation, implying a possible common ancestor.

All described mutations are novel and cause a stop mutation, leading to a truncated protein. Neither of the variants reported in this study represent a frequent polymorphism according to the Genome Aggregation Database (9). Apart from the abovementioned families I and II, all reported families outside the Amish community harbor different mutations (4, 7, 8): the reported four European families with *SPG21* (Austria, Germany, and Italy) (3, 8) all show truncating mutations like those in the Amish families (4, 8). The Asian family, however, displayed missense mutations (7). Differences in the type of mutation may account for distinct phenotypes: The Japanese patients reported by Ishiura showed late disease onset and did not exhibit cerebellar, extrapyramidal, or bulbar signs (7). Our families exhibited predominant spastic para- or tetraparesis with pyramidal signs, pronounced cognitive impairment, ataxia, and extrapyramidal signs (chorea, athetosis, and dystonia) (Table 1). Extrapyramidal involvement was reported mainly in the Amish cohort with predominant chorea, athetosis, and oromandibular dyskinesias (4, 6). Furthermore, dystonic posturing was noted in one patient by Scarlato et al. (8). In addition to the features reported above, subject I-1 exhibited axonal-sensorimotor neuropathy, and subject III-1 first became symptomatic with paranoid psychosis 5 years before the onset of motor symptoms. While retinal changes were not described as part of the original syndrome, subject III-2 showed retinitis pigmentosa. Taken together, the clinical presentation of our patients is largely comparable to that of the previously published Amish families (4, 6, 8). There was, however, a difference in age at onset. Apart from the Japanese family, the typical onset of disease was described to be from childhood to adolescence (4, 6). The reported subjects here, however, show variable ages at the onset of disease. As age of onset in HSP may range from the first year of life to the seventies (3), it seems plausible that SPG21 is under-diagnosed especially in patients with onset after the second decade of life. Our report, thus, highlights the importance of considering SPG21 in patients with an according phenotype despite an onset later in life.

Taken together, this report further extends the clinical phenotype of *SPG21*. Our study expands *SPG21* to the middle European population and indicates that mutations in the *ACP33* gene should be considered in non-Amish populations.

## Data Availability Statement

The data analyzed in this study is subject to the following licenses/restrictions: Clinical data, anonymized data can be shared upon request. Requests to access these datasets should be directed to Matthias Amprosi, matthias.amprosi@i-med.ac.at; Wolfgang Nachbauer, wolfgang.nachbauer@i-med.ac.at.

## Ethics Statement

Ethical review and approval was not required for the study on human participants in accordance with the local legislation and institutional requirements. Written informed consent for participation was not required for this study in accordance with the national legislation and the institutional requirements.

## Author Contributions

MA, EI, WN, AH, CS, AE, CG, SB, and TK performed the recruitment and clinical evaluation of the patients. MA, WN, AH, SB, and TK conceptualized the project and interpreted the clinical data. MA, EI, WN, AH, CS, CG, SB, and TK wrote the first draft of the manuscript. WN, SB, and TK supervised the project. All authors read the manuscript, contributed to manuscript revision, and approved the final version.

## Conflict of Interest

The authors declare that the research was conducted in the absence of any commercial or financial relationships that could be construed as a potential conflict of interest.

## Publisher's Note

All claims expressed in this article are solely those of the authors and do not necessarily represent those of their affiliated organizations, or those of the publisher, the editors and the reviewers. Any product that may be evaluated in this article, or claim that may be made by its manufacturer, is not guaranteed or endorsed by the publisher.
